# Shall Physicians Dispense Their Own Medicine?

**Published:** 1881-07

**Authors:** M. G. Pingree


### SHALL PHYSICIANS DISPENSE THEIR OWN MEDICINE?

By M. G. PINGREE, M.D.

(Abstract of a Paper read before the Chicago Medical and Surgical Society.)

Although members of a noble profession, we are all men and women of the world. Our chances for the observation and study of human nature are unequaled, and we have *all* found, sad as it may seem, that the physician is bound and controlled by the same leveling and corrupting influences as is the balance of humanity. From the over-zealous apostle of truth, we are constantly suspicious of a breach of trust, and when we gaze upon a doctor who noisily preaches in favor of a higher medical education, who advocates the immediate weeding out of any and all vicious quacks and soulless charlatans, and who affirms, with vehement iteration, that *he* practices solely for the benefit of suffering humanity, we see a liar and a hypocrite. Do not understand me to say that no man or woman practices medicine for a love of it, but that they as a class do so for the sole benefit of the afflicted, I emphatically deny. Why is it that our noble profession is dwarfed and corked by such a vast shower-bath of sickly sentimentalism? The practice of medicine is as much a purely business transaction as is the vending of groceries and hardware, and the sooner we, as Eclectics, preach and *practice* upon this basis, the sooner we shall be looked upon as genuine reformers, and the greater will be the respect and confidence of the public. Naturally enough, we would inevitably gain a large share of its patronage. You may say, "Why, doctor, all of this is not germane to the subject in hand;" but I say it is, for it is from a pecuniary standpoint that I wish principally and briefly to argue in the affirmative of the question, "Shall doctors dispense their own medicines?" For centuries people have heard so often the refrain of "noble profession, lofty profession, sacred profession, God-like profession," etc., as applied to the calling of medicine, that we have entirely lost sight of the practical and prosaic side of the business, which is really the most important to us, as it involves the fact of wealth or poverty, position or obscurity, comfort or the indefinable dread of a moneyless man. The fact is indisputable, that the vast majority of mankind regulate their business affairs upon business principles. Other things being equal, they purchase their supplies where they think they can get them the best and cheapest. Doctors will not admit that people carry this idea into their relations with the medical profession, but in this, as in many other things, they are sadly mistaken. A patient calls upon a doctor (eclectic or allopathic), who dispenses his medicines solely through the medium of druggists. He has a cough, and wants something to cure it. We write a prescription, and charge him $1 for the same. He then goes to the drug store and has it compounded, for which he pays an additional 50 or 75 cents. Here is a palpable injustice to the patient. On the other hand, a friend or friends of his have similar coughs. He says, "Here is a bottle which contained a cough mixture prescribed for me by Dr. A. or Dr. B. It cured my cough at once. Take it to the drug store and have it re-filled." Here is a palpable injustice to the doctor. In the first instance, the patient is defrauded, in the second the doctor is robbed. In both instances the druggist is the gainer. I am personally conversant with the fact that prescriptions of my own have been re-filled, without my knowledge or consent, from twice to fifteen times, and have gone into as many different families. The patron of homœopathy goes to his physician, who, instead of writing a prescription, puts up a package of medicine from his own stock, and sends the patient off rejoicing, minus $1. If the patient is cured, he does not send his friends to the druggist for similar medicine, but to the doctor that cured him. I have, for many years, felt confident that the marvelous growth of homœopathy depended, in a great measure, upon its greater economy. I believe that were it generally known that eclectic physicians dispensed their medicines, they would leap at once to the front in popular favor, and soon outstrip other schools. Much has been said, and much more will be said, pro and con, in regard to this subject, but I verily believe that no man or woman ever lived to practice medicine, and wrote more than a dozen prescriptions, who has not had his or her prescriptions repeated and re-filled without their knowledge or consent. No legislation can be devised that can ever blot out this evil. The remedy is in our own hands—that is, dispense our own medicines.

Perhaps you will say, "Druggists must live, as well as doctors." Very true. But, right here, comes to my aid the inexorable law of the "survival of the fittest." Why should druggists enrich themselves at our expense? If there are too many druggists, the fittest will undoubtedly survive, and the balance can find other legitimate occupations. I can readily understand how difficult it would be for any physician to dispense all the remedies that he uses in the treatment of chronic diseases, for, in these cases, are necessitated such bulky remedies as cod liver oil, extract of malt etc.; but in the treatment of acute diseases there is absolutely no need of writing prescriptions. In addition to the greater economy to the patient, the physician derives greater satisfaction from his treatment, and is seldom disappointed in the therapeutical action he desires from the remedies given. I will cite a single instance to demonstrate the truth of these statements: Two weeks ago, a gentleman, who is employed in a jewelry house at a moderate salary, called at my office for advice. Upon examination, I found his tonsils badly inflamed and swollen, eyes congested, face flushed, very severe and distressing headache, pulse 120, alternate chills and fever, etc., indicating a typical case of tonsilitis. I gave this patient three doses of jaborandi, and fifteen doses of aconite, directing him to use the former until free perspiration ensued, and follow it up with the latter. He asked me what my fee was, and upon being told $1, his eyes fairly shone with pleased astonishment. "Why," said he, "you shall *always* doctor me after this." The next day he reported himself to me as being entirely well. He was a "transient," and had been in the habit of paying a doctor $1 for advice, and as much more for medicine. The medicine I gave him would have cost me 5 cents. How much better it is to have ten patients at a profit of 95 cents each, than five patients at a profit of $1 each. I have great charity for many of the thousands of poor people who, although they seek our services, refuse to liquidate their indebtedness to us. After a man who has been earning from $1.50 to $2 per day, arises from a bed of sickness of from two to four weeks' duration, and is confronted with a doctor's bill for from fifteen to thirty visits, at $2 a visit, and a druggist's bill for half as much more, he simply despairs of liquidating it, and so lets it go, and the next time he is sick sends for some other doctor. It is not because he is dishonest, but it is utterly impossible to pay, unless at the sacrifice of many of the necessaries of life. I have noticed this one fact, *i. e.*, when the doctor's bill is $25, and the druggist's bill is $10, the druggist invariably gets his money first. When a doctor refuses to pay a visit to a man because he cannot pay, the public and press at once raise a prolonged howl of indignation and cry "shameless, heartless, brutal," etc., *ad nauseum.* But when the druggist refuses to trust a poor, starving and sickly wretch for a bottle of medicine, he is never condemned, but receives the credit of conducting his business upon sound business principles. Why the difference? Simply because physicians themselves have so invested the practice of medicine with the glamour of sentimentalism that the public have come to believe them. The practice of medicine is not a sentiment but a business. We study and plan and think how we best can benefit our patients, because we feel that the amount of our business depends upon the success with which we dispense our remedies, and that one patient well cured is a walking advertisement of our skill, whilst a patient not cured, or let die, reflects no credit upon us. In other words, we endeavor to retail a first-class article of professional merchandise, in order that others may come to our market for similar wares. Let us institute a beginning of reform, and let us lay, as an enduring foundation, the principle and *practice* of dispensing our own medicines.—*Chicago Medical Times.*

